# Dialkyl Carbonates in the Green Synthesis of Heterocycles

**DOI:** 10.3389/fchem.2019.00300

**Published:** 2019-05-07

**Authors:** Pietro Tundo, Manuele Musolino, Fabio Aricò

**Affiliations:** ^1^Department of Environmental Sciences, Informatics and Statistics, Ca' Foscari University, Scientifico, Venice, Italy; ^2^Institute for the Chemistry of Organometallic Compounds (ICCOM), National Research Council of Italy (CNR), Florence, Italy

**Keywords:** heterocycles, green synthesis, cyclization, dialkyl carbonates, green chemistry

## Abstract

This review focuses on the use of dialkyl carbonates (DACs) as green reagents and solvents for the synthesis of several 5- and 6-membered heterocycles including: tetrahydrofuran and furan systems, pyrrolidines, indolines, isoindolines, 1,4-dioxanes, piperidines, and cyclic carbamates. Depending on the heterocycle investigated, the synthetic approach used was different. Tetrahydrofuran systems, pyrrolidines, indolines, isoindoline, and 1,4-dioxanes were synthesized using dimethyl carbonate (DMC) as sacrificial molecule (B_Ac_2/B_Al_2 mechanism). Cyclic carbamates, namely 1,3-oxazin-2-ones, were prepared employing DACs as carbonylating agents, either by B_Ac_2/B_Al_2 mechanism or through a double B_Ac_2 mechanism. Piperidines were synthetized taking advantage of the anchimeric effect of a new family of dialkyl carbonates, i.e., mustard carbonates. Finally, in the case 5-hydroxymethylfurfural (HMF), DMC has been employed as efficient extracting solvent of this extensively investigated bio-based platform chemical from the reaction mixture. These synthetic approaches demonstrate, once again, the great versatility of DACs and their—yet to be fully explored—potential as green reagents and solvents in the synthesis of heterocycles.

## Introduction

Dialkyl carbonates (DACs) are well-known and extensively exploited safe, green reagents and solvents. In particular, dimethyl carbonate (DMC)—the smallest organic carbonate—encompasses the following desirable features of a green compound (Tundo and Selva, 2002; Tundo et al., 2018):

### Green Synthesis

**Scheme 1:**
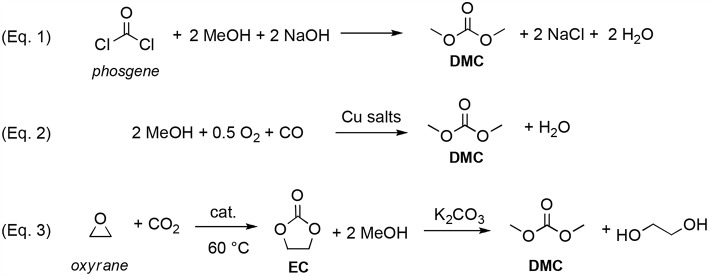
Syntheses of DMC. Via phosgene (Equation 1); by catalytic oxidative carbonylation of methanol (Equation 2); by CO_2_ insertion into oxyrane (Equation 3).

DMC was initially produced by reaction of phosgene with methanol in basic conditions (Equation 1; Scheme 1). However, this process caused also the formation of a considerable amount of NaCl that had to be disposed of (Shaikh and Sivaram, 1996).

The main turning point in DACs exploitation was the development by Enichem (Romano et al., 1979) and UBE (Nishihira et al., 1993), of a greener synthesis of DMC, based on the catalytic oxidative carbonylation of methanol with oxygen (Equation 2; Scheme 1). As a result, since the middle 80's, DMC was produced in a commercial scale and started to be investigated as reagent and solvent in several industrial processes.

Over the years, numerous other green approaches to DMC were reported including alcoholysis of urea (Ball et al., 1980; Wang et al., 2005; Wu et al., 2012) and direct conversion of CO_2_ and methanol into DMC employing inorganic and organic dehydrating agents (Yamazaki et al., 1979; Hoffman, 1982; Zhang et al., 2011; Fan et al., 2012). Nowadays DMC is mainly produced by insertion of CO_2_ into oxyrane to give ethylene carbonate (EC), which—by reaction with methanol in basic conditions—generates DMC and ethylene glycol (Equation 3; Scheme 1) (Kondoh et al., 1995; Miyaji et al., 2007; He et al., 2014; Wang et al., 2014; Martín et al., 2015).

### No Toxicity

DACs display only low (eco)toxicity and are completely biodegradable. In particular, DMC is classified as a flammable liquid that does not smell (methanol-like odor) and does not have irritating or mutagenic effects by either contact or inhalation (Lamoureux and Agüero, 2009). As a result, DMC is a safe to handle compound and a very efficient alternative to chlorine reagents, as it can replace toxic methyl halides and dimethyl sulfate in alkylation reactions and phosgene in alkoxycarbonylation reactions. Furthermore, it can be employed as a substituted of halogenated solvents as dichloromethane and chloroform.

### Numerous Applications as a Solvent

Dialkyl carbonates offer numerous vantages as solvents since they are stable under ambient conditions and available in large amounts at low prices. Besides DACs polarity and boiling points can be easily tuned by varying length and nature of the alkyl groups.

DACs are considered aprotic highly dipolar solvents like DMSO or DMF, although they show (except DMC) only limited miscibility with water.

The use of organic carbonates as solvents for electrochemical applications has been extensively investigated especially as a non-aqueous electrolyte. Furthermore, their use in extractive procedures is also well-established. In recent years, DACs are also started to be investigated as possible alternatives to replace VOCs and as co-solvents in cleaning processes and in cosmetics (Schmeidl, 1990; Rüsch gen. Klaas and Warwel, 2001; Su et al., 2009; Schfäfner et al., 2010; Olschimke et al., 2012; Tannir et al., 2012; Huang et al., 2015).

### Versatility as Green Reagent

**Scheme 2:**
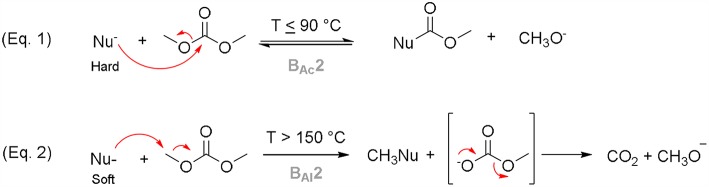
DMC reactivity according to the HSAB theory.

DACs are versatile reagents that can be employed both in alkylation (instead of alkyl halides) and alkoxycarbonylation reactions (instead of phosgene) (Climent et al., 2011; Tundo et al., 2018). In general, organic carbonates chemistry follows the Pearson's Hard-Soft Acid-Base (HSAB) theory (Pearson, 1963). In presence of a base and at *T* < 90°C, a DAC can react with a hard nucleophile at the sp^2^ carbonyl moiety via bimolecular base-catalyzed acyl-cleavage (B_Ac_2) substitution (Equation 1; Scheme 2) (Tundo et al., 2009; Grego et al., 2012). On the other hand, if the reaction is conducted at *T* > 150°C, DACs generally react—as alkylating agents—with a soft nucleophile at the saturated sp^3^ carbon via bimolecular base-catalyzed alkyl-cleavage (B_Al_2) substitution (Equation 2; Scheme 2) (Tundo et al., 2009; Aricò and Tundo, 2016b). DACs reactivity has been investigated with numerous monodentate and bidentate nucleophiles (Rosamilia et al., 2008a,b; Fiorani et al., 2018) in batch as well as in continuous-flow apparatus (Tundo et al., 2010c; Grego et al., 2013).

Another example of DACs versatility is the use of DMC for biocatalytic synthesis of glycerol carbonate. In this catalytic route glycerol was reacting with DMC in the presence of lipase under solvent-free conditions (Tudorache et al., 2012; Cushing and Peretti, 2013).

However, HSAB theory is not exhaustive in explaining all the features of DACs reactivity. The use of DACs in the preparation of heterocyclic compounds is a poignant example in which factors, such as entropic or anchimeric effects may drive the reaction mechanism. In this prospect, the purpose of this review is to report the use of DACs as efficient reagents and solvents in the green synthesis of five- and six-membered heterocycles.

The versatility of the organic carbonates is pivotal in these cyclization reactions, inasmuch, depending on the target heterocycle, DACs may be used as sacrificial molecule, carbonylating agent, reaction media, promoter of ring expansion or transposition reactions.

## 5-Memebered Heterocycles

### Tetrahydrofuran and Furan Systems

Tetrahydrofuran systems are incorporated as structural subunits in numerous natural and synthetic compounds, such as muscarine (Matsumoto et al., 1969), lithospermic acid (Wang and Yu, 2011), obtusafuran, kadsurenone (Benbow and Katoch-Rouse, 2001), polyether antibiotics (Westley, 1982), inostamycins (Imoto et al., 1990), etc.

The simplest method for the synthesis of tetrahydrofurans is via acidic cyclodehydration of 1,4-diols, i.e., the preparation of tetrahydrofuran from 1,4-butanediol (Olah et al., 1981; Pinkos et al., 2004; Mitsudome et al., 2012). In the literature, there are reported several other synthetic approaches to cyclic ethers, such as cyclization or cycloaddition reaction. These procedures generally employ heavy metals (Sharma et al., 2002; Shibata et al., 2005; Panda and Sarkar, 2008; Gadda et al., 2010; Tsui et al., 2012) or chlorine chemistry in the form of leaving groups, i.e., tosylate, mesylate etc. (Grubb and Branchaud, 1997; Lindner and Rodefeld, 2001; Adaligil et al., 2007).

**Scheme 3:**
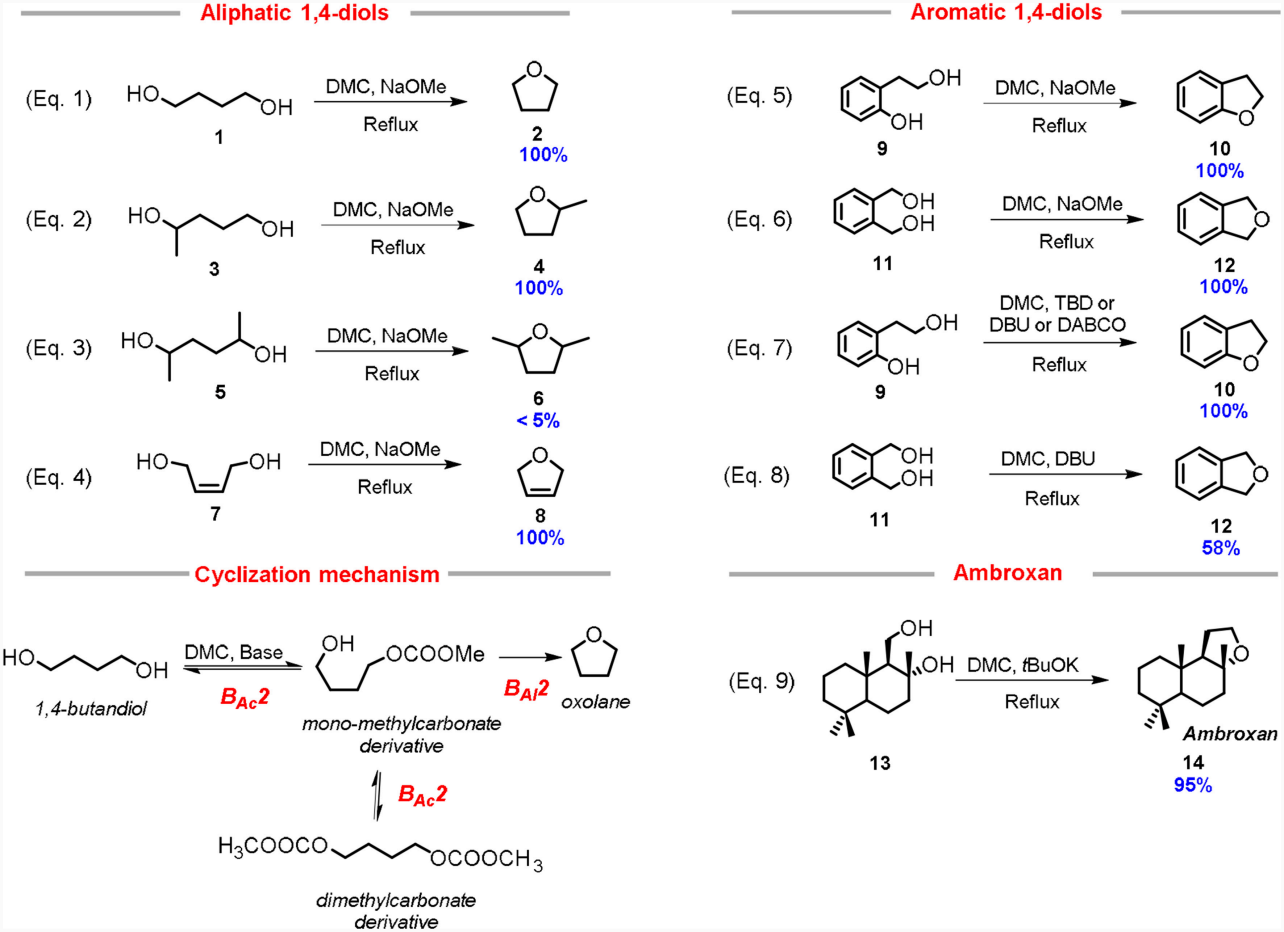
Synthesis of 5-membered aliphatic and aromatic heterocycles via DMC chemistry. Reaction conditions: (Equation 1): **1**: NaOMe: DMC in 1.0: 2.0: 4.0 molar ratio in ACN, *T* = 60°C, *t* = 4 h; (Equation 2): **3**: NaOMe: DMC in 1.0: 3.0: 4.0 molar ratio in ACN, *T* = 70°C, *t* = 6 h; (Equation 3): **5**: NaOMe: DMC in 1.0: 3.0: 4.0 molar ratio in ACN, *T* = 70°C, *t* = 24 h; (Equation 4): **7**: NaOMe: DMC in 1.0: 2.0: 4.0 molar ratio in ACN, *T* = 70°C, *t* = 4 h; (Equation 5): **9**: NaOMe: DMC 1.0: 2.0: 4.0 molar ration in ACN, *T* = 70°C, *t* = 2 h; (Equation 6): **11**: NaOMe: DMC in 1.0: 2.0: 4.0 molar ratio in ACN, *T* = 70°C, *t* = 4 h; (Equation 7): **9**: DMC: base 1.00: 4.00: 0.05 molar ratio, *T* = 90°C, *t* = 8 h; (Equation 8): **11**: DMC: DBU 1.0: 8.0: 1.0 molar ratio, *T* = 90°C, *t* = 24 h; (Equation 9): Amberlyn **13**: *t*BuOH: DMC in 1.0: 2.0: 30.0 molar ratio, *T* = 90°C, *t* = 3 h.

Advances in the research of greener approaches to heterocyclic structures have been achieved by using alternative reagents. In this view, it has been reported that 5-membered cyclic ethers can be easily synthesized starting from1,4-diols by DMC chemistry in mild conditions and high yield. In particular, tetrahydrofuran (THF) was synthesized in a quantitative yield by reacting 1,4-butanediol with DMC (10.0 mol eq.) using a stoichiometric excess of a strong base, such as NaOMe or *t-*BuOK (2.0 mol eq.) and heating this solution to reflux for 4 h (Equation 1; Scheme 3) (Aricò et al., 2012c).

The cyclization of 1,4-butanediol **1** to THF **2** can be ascribed to the versatility of the DMC as reagent, that operates as a sacrificial molecule (Equation 1; Scheme 3). In fact, most likely, the cyclization mechanism comprises of the 1,4-diol methoxycarbonylation by a B_Ac_2 mechanism, followed by an intramolecular cyclization thorough a B_Al_2 mechanism with the release of the methylcarbonate anion as leaving group (Cyclization mechanism, Scheme 3).

Several other substrates were tested so to investigate the general applicability of this synthetic approach. 2-Methyl tetrahydrofuran **4** was prepared from 1,4-pentanediol **3**, in high yield under similar conditions, although a quantitative conversion required an excess of base (3 mol eq.) to cope with the lower reactivity of secondary alcohols (Equation 2; Scheme 3). In another example, the reaction performed using 2,5-hexandiol **5** (mixture of stereoisomers) gave the corresponding cyclic ether **6** only in traces (< 5%); the substrate conversion was moderate even after 24 h (Equation 3; Scheme 3). In this case, even by using an excess of base (3.0 mol eq.), the cyclization was hindered by the reduced reactivity of secondary alcohols, both in the methoxycarbonylation step and in the nucleophilic substitution reaction. This latter result demonstrates that the DMC-based cyclization reaction requires to take place at least one primary alcohol incorporated in the starting diol. In fact, *cis*-1,4-but-2-ene diol **7** underwent fast and quantitative cyclization reaction (Equation 4; Scheme 3). In this case study, the formation of the related 2,5-dihydrofuran **8** is probably aided by the favorable *cis* position of the primary alcohol moieties; as a matter of fact, when the reaction was performed with a catalytic amount of sodium methoxide (5 mol %) the cyclic compound still formed in appreciable yield (30% yield).

The reactivity of 1,4-diols bearing aromatic moieties, i.e., 1,2-dihydroxymethyl benzene **11** and 2-hydroxyethyl phenol **9** was also reported. 2-Hydroxyethyl phenol **9** demonstrated to be a very reactive 1,4-diol, in fact, 2,3-dihydrobenzofuran **10**, was the only product formed even in the presence of substoichiometric amount (0.5 mol eq.) of a base (Equation 5; Scheme 3).

1,2-Bis(hydroxymethyl)benzene **11** also led to the quantitative formation of phthalan **12** under similar reaction conditions (Equation 6; Scheme 3).

Some preliminary computational studies on this DMC-based cyclization reaction were then carried out. It was showed that after methoxycarbonylation of one hydroxy groups (reactivity order: primary alcohol > secondary alcohol > tertiary alcohol) the pathway leading to the cyclic ethers was the most energetically favored (Aricò et al., 2012c, 2018b).

This *in silico* evidence confirmed the proposed two-step mechanism for the DMC-assisted cyclization (Scheme 3) involving the methoxycarbonylation of the less hindered alcohol via B_Ac_2, followed by an intramolecular alkylation reaction via B_Al_2. The former step follows the HSAB theory, while the latter is promoted by high entropic effects leading to ring closure.

Recent investigations by our research group showed that a nitrogen bicyclic base is effective for the one-pot synthesis of heterocycles from aromatic 1,4-diols also when used in a catalytic amount (Aricò et al., 2015a). Nitrogen bicyclic bases, such as 1,8-diazabicyclo[5.4.0]undec-7-ene (DBU), 1,4-diazabicyclo[2.2.2]octane (DABCO) and triazabicyclodecene (TBD) demonstrated to enhance the reactivity of DMC in methoxycarbonylation reaction by B_Ac_2 mechanism (Carafa et al., 2011; Quaranta et al., 2014).

In the case of 2-hydroxyethyl phenol **9**, the DMC-mediated cyclization reaction was promoted by a catalytic amount of several nitrogen bicyclic bases, i.e., DBU, DABCO, and TBD. All those bases resulted efficient catalysts when used in 5% mol eq., providing quantitative conversion and selectivity toward the 2,3-dihydrobenzofuran **10** (Equation 7; Scheme 3) Furthermore, it was possible to perform the cyclization in almost neat conditions by reducing the DMC consumption up to 4.0 mol eq. relative to the starting 1,4-diol (Aricò et al., 2015a).

The cyclization of 1,2-bis(hydroxymethyl)benzene **11** was then tested in similar reaction conditions, however none of the bicyclic nitrogen bases led to the quantitative synthesis of the related cyclic, 1,3-dihydroisobenzofuran **12**. The best yield (58%) was achieved employing DBU as catalyst (Equation 8; Scheme 3). This result is probably due to the lower acidity of the hydroxy group incorporated into the substrate **11** compared to the more reactive phenol unit of the previously investigated 2-(2-hydroxyethyl)phenol **9** (Aricò et al., 2015a).

An interesting application of the DMC-mediated cyclization reaction is the preparation of ambroxan **14**, also called (-)-norlabdane oxide, by using as starting reagent amberlyn diol **13** (Equation 9; Scheme 3). Ambroxan is used for providing ambergris-type odors to perfumes, since the natural ambergris is no longer available (Steenkamp and Taka, 2009; Bevinakatti et al., 2013). Results showed that amberlyn diol **13** cyclized quantitatively to ambroxan within only 3 h, using two equivalents of potassium *tert*-butoxide in an excess of DMC (used as reagent and solvent). The amount of DMC could be reduced without affecting the outcome of cyclization as confirmed by performing the reaction in THF in the presence of three equivalents of DMC. Furthermore, it is noteworthy that the reaction via DMC preserves the chiral integrity of the starting material in the product.

In conclusion, it could be stated that, compared to commonly used chlorine-based procedure, the synthesis of heterocycles incorporating a tetrahydrofuran unit via DACs chemistry and under basic conditions is high-yielding, does not require chlorine chemicals or inorganic acids and the products can be isolated without employing any time-consuming purifications. This procedure can be considered of general application, since it has been proven efficient for aliphatic and aromatic 1,4-diols. The only limitation is that the starting diols should incorporate at least one primary hydroxy group, in order to render the cyclization effective.

**Scheme 4:**
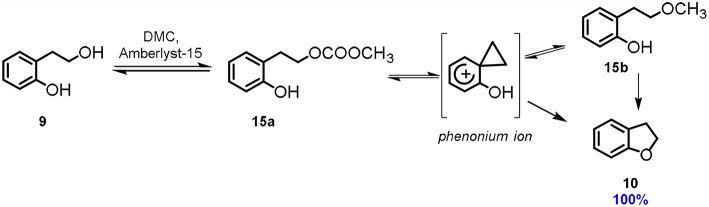
Cyclisation via phenonium ion. Reaction conditions: **9**: DMC 1.0: 60.0 molar ratio, Amberlyst-15 (100% w/w referred to **9**), *T* = 90°C, *t* = 48 h.

A different synthetic approach to 2,3-dihydrobenzofuran **10** via DMC chemistry was reported using acidic conditions, instead of basic ones (Aricò et al., 2018b). Several acids, organic and inorganic, have been tested for the cyclization of 2-(2-hydroxyethyl)phenol **9**. Among them, the exchange resin Amberlyst-15 resulted by far the most efficient. 2-(2-Hydroxyethyl)phenol **9** is an interesting substrate, as the 2-hydroxyethyl moiety is located in *ortho* position to the aromatic hydroxy group and thus it is capable of promoting the phenonium ion formation (Scheme 4).

Theoretical calculations suggested that the most favorable reaction pathway is the formation of a phenonium ion via DMC chemistry that is then converted into the 2-(2-methoxyethyl)phenol **15b**. 2,3-Dihydrobenzofuran **10** is finally formed via intramolecular cyclization of this intermediate.

It is noteworthy that in this peculiar acid catalyzed cyclization reaction, DMC is used as solvent, methoxycarbonylation agent as well as the leaving group promoting the phenonium ion formation.

Another interesting example of green synthesis of furan system via DMC chemistry is the preparation of bio-based platform molecule 5-hydroxymethylfurfural **17** (HMF). HMF is an archetype of the widely investigated furan-based platform molecules (Bozell and Peterson, 2010; Ruppert et al., 2012). This compound has found numerous applications as a building block in the synthesis of chemicals, materials, bio-based polymers and fuels (Mika et al., 2018). As a result, numerous synthetic procedures to HMF have been reported in the literature, which mostly rely upon the acid-catalyzed triple dehydration of D-fructose (Karinen et al., 2011; Qiao et al., 2015).

**Scheme 5:**
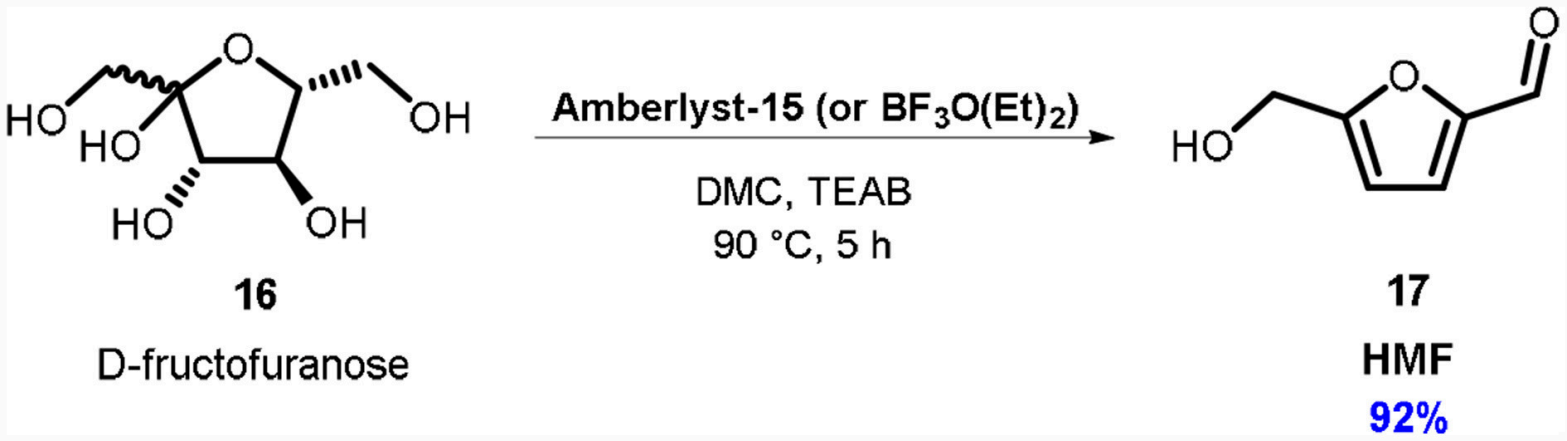
Conversion of D-fructose **16** into HMF **17**. Reaction condition: **16**: TEAB: DMC in 1.00: 0.17: 16.80 molar ratio, Ambelyst-15 or BF_3_O(Et)_2_ (10% w/w referred to D-fructose), *T* = 90°C, *t* = 5 h.

Compared to several of these preparations of HMF, the DMC-based one uses only commercially available materials and had a very simple work-up (Scheme 5). In the best-found reaction conditions, D-fructose **16** (1.0 eq. mol) was dissolved in a solvent system consisting in a mixture of DMC and TEAB (99:1 mol ratio) in the presence of Amberlyst-15 or Lewis acid BF_3_O(Et)_2_ (10% weight) as a catalyst (Musolino et al., 2018).

In this novel reaction media, TEAB favors the dissolution of D-fructose, meanwhile DMC acts as an efficient extraction solvent. As a result, HMF **17** can be recovered as a pure product from the reaction mixture in > 90% yield by simple evaporation of the DMC. Large-scale preparations of HMF (up to 20 grams of D-fructose) were also conducted, without affecting the almost quantitative yield of this synthetic approach.

Materials efficiency performance of the HMF preparation via DMC was also taken into consideration; E-factor and PMI green metrics were calculated. The results were compared to literature reports for the same dehydration reaction, where selection criteria included isolation of HMF and the reaction scale was at least one-gram. In fact, although in the literature there are reported numerous synthetic procedures to HMF, most of them relies upon analytic techniques, such as high performance liquid chromatography (HPLC), for determining HMF yield. This is due to the difficult separation of the product from the reaction mixture and to the well-know HMF instability (Musolino et al., 2018). To the best of our knowledge, the synthetic procedure using DMC as extracting solvent resulted so far, the most convenient among the ones investigated. However, it should be pointed out, that, due to its complexity, there is still much room for improvement in terms of E-factor contributors for this reaction.

### Cyclic Sugars: Isosorbide and Isomannide

Over the last 10 years, anhydro sugar alcohols held a top position in the biorefinery development. Among them, isosorbide **18** and isomannide **19** both encompass all of the desired criteria for a bio-based platform. In fact, these compounds have found many applications in food industry, pharmaceutical field, and biopolymer preparation (Bozell and Peterson, 2010).

Furthermore, alkyl derivatives of isosorbide **18**, such as dimethyl isosorbide have been investigated as potential substitutes of high-boiling solvents, such as dimethyl sulfoxide (DMSO) and dimethylformamide (DMF) (Aricò et al., 2017).

**Figure 1:**
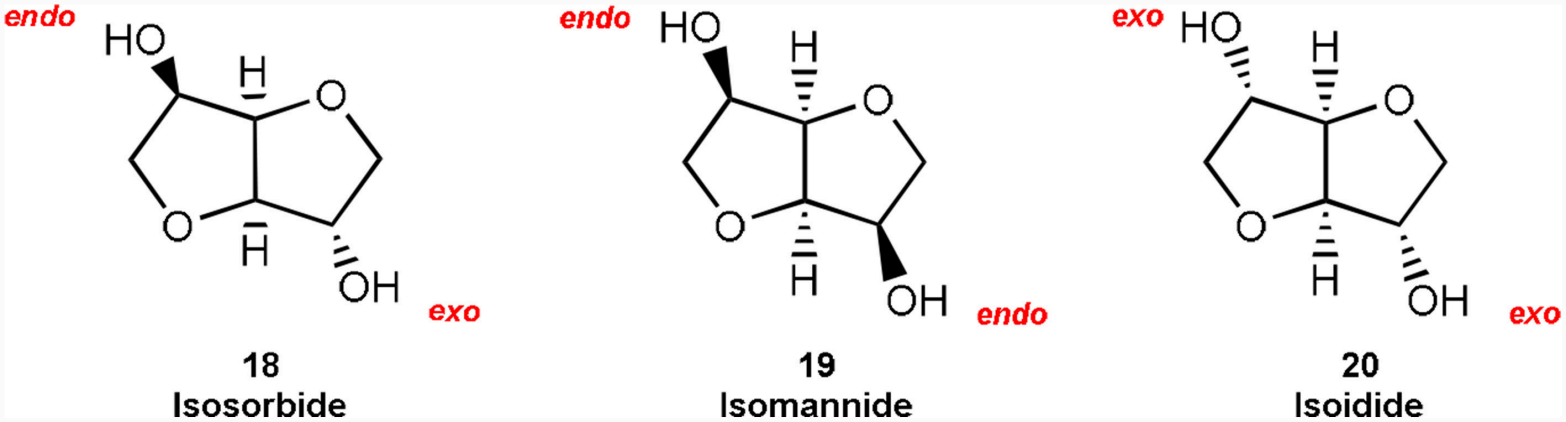
Chemical structure of isosorbide and its epimers isomannide and isoidide.

Isosorbide **18** is also well-known for having a high and unexpected reactivity, probably due to its peculiar open-book configuration formed by two *cis-*fused tetrahydrofuran rings, where the four oxygens incorporated in the structure are in β-position to each other. The configuration of the two hydroxy groups—one *endo* (2-position) directed toward the V-shaped cavity, and the second *exo* (5-position) pointing outside of the sugar cavity (Figure 1)—has been showed to influence the reactivity of isosorbide **18**. In fact, isoidide **20** and isomannide **19** (Figure 1), that incorporate either *exo* or *endo* hydroxy groups, showed a diverse reactivity.

Isosorbide and its epimer isomannide are industrially synthesized from their parent alcohols D-sorbitol **17** and D-mannitol respectively by a double dehydration reaction, using different types of catalysts (Rose and Palkovits, 2012). The actual synthetic approach to cyclic sugar isosorbide is focused on less toxic and easy to recover heterogeneous acidic catalysts, such as mixed oxides, phosphated or sulphated oxides, sulphonic resins, and bimetallic catalysts (Aricò and Tundo, 2016a). However, despite these new and promising methodologies, the main issue remains the separation and purification of isosorbide from the reaction mixture that might contain elimination products or other cyclization intermediates, such as 1,4-sorbitan derivatives.

**Figure 2:**
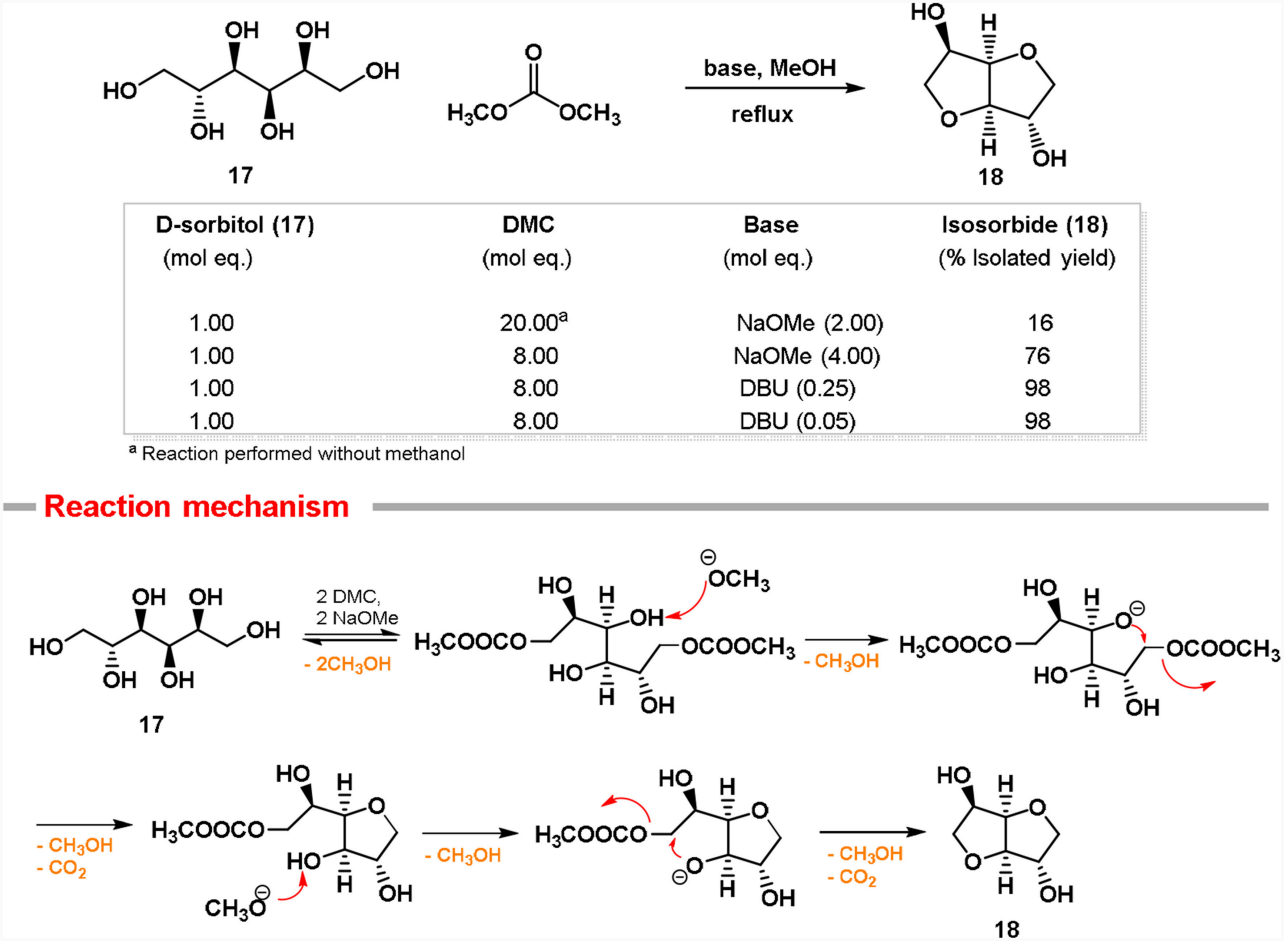
Conversion of D-sorbitol **17** into isosorbide **18** via DMC chemistry.

In this prospect, a DMC-based synthetic approach has been exploited to achieve isosorbide (Figure 2). The idea was to employ DMC as a dehydrating agent in mild reaction conditions (Tundo et al., 2010a). In a first set of experiments, D-sorbitol **17** was dissolved in DMC (20 mol eq.) and reacted at 90°C using an excess of strong base (2.0 mol eq.), i.e., sodium methoxide. In these conditions, isosorbide **18** was isolated only in modest yield (16%), as once it is formed, it further reacted with DMC leading to the formation of its methoxycarbonyl and methyl derivatives. In order to prevent further reactions, methanol was added to the reaction mixture as a co-solvent. By using this synthetic procedure, the numerous equilibria that affect the reaction can be efficiently shifted toward the isosorbide formation, preventing any by-products production. The best result, i.e., 76% isolated yield of isosorbide, was achieved when an excess of NaOMe was employed (4.0 mol eq.). The use of an excess of base has to be ascribed to the complexity of this one-pot double cyclization reaction that requires two equivalents of base for each tetrahydrofuran formed (see reaction mechanism in Figure 2).

Recently, we also reported that bicyclic nitrogen base DBU can be used in the high-yielding synthesis of isosorbide via DMC chemistry. The main advantage of this synthetic approach is that DBU works efficiently in substoichiometric amount (0.25 mol eq.) (Aricò et al., 2015a). In these reaction conditions, isosorbide can be isolated as a pure compound by a simple filtration on a silica pad, followed by DMC evaporation. Even when the amount of DBU was reduced to 5% mol eq., the wanted cyclic sugar was still formed in a quantitative yield.

This synthetic approach can be also employed for the cyclization of D-mannitol into isomannide **19**. It is noteworthy that both anhydro sugars isosorbide and isomannide are formed at low temperature (90°C) through a one-pot double cyclization reaction, thus the amount of DBU is 2.5% mol eq. for each tetrahydrofuranic cycle formed and in turn it can be considered as a catalytic amount.

Compared to previous works reported in the literature on isosorbide and isomannide preparation (Bozell and Peterson, 2010), the DMC-based synthetic methodology is one-pot, environmentally friendly (DMC can be eventually recycled), does not require any purification, allows very pure products and does not affect the chiral integrity of the substrates. To the best to our knowledge, the DMC-based approach is the highest yielding preparation reported so far for cyclic sugars.

### Pyrrolidines, Indolines and Isoindolines

Five-membered *N*-heterocycles are present in numerous natural products, i.e., vitamins, hormones, and alkaloids (Padwa and Bur, 2004). As an example, pyrrolidines are often incorporated as key units in pharmaceuticals, herbicides, pesticides and dyes (Katritzky et al., 2010).

Similarly, to the 5-membered *O*-heterocycles above discussed, pyrrolidine systems can be synthesized employing DMC as a sacrificial molecule (Aricò et al., 2012b). In this view, both aliphatic and aromatic substrates have been investigated.

Preliminary experiments were conducted on 4-amino-1-butanol **21** using DMC as solvent and reagent in the presence of several catalysts (10% mol eq.); examples include metallic homogenous catalysts (Entry 1-2; Table 1), alkali and heavy metal basic carbonates (Entry 3-5; Table 1), strong bases (Entry 6-7; Table 1) and hydrotalcites (Entry 8; Table 1). All the reactions were carried out in autoclave at 180°C and they resulted in the formation of the wanted *N*-methoxycarbonyl pyrrolidine **22** in good yield (up to 62%) when cesium carbonate was used as a base (Entry 4; Table 1).

**Table 1:** Synthesis of *N*-methoxycarbonyl pyrrolidine using different bases/catalysts^a^.

**Entry**	**Base/catalyst**	**eq**	***N*-methoxycarbonyl pyrrolidine 22 (%)^b^**
1	ZnO(Ac_2_)	0.1	35
2	SnOBu_2_	0.1	28
3	K_2_CO_3_	0.1	49
4	Cs_2_CO_3_	0.1	62
5	(ZnCO_3_)_2_·[Zn(OH)_2_]_3_	0.1	39
6	MeONa	0.1	46
7	*t*BuOK^c^	0.5	76^d^
8	HT KW2000^e^	n.a.	48

a Reaction conducted in autoclave heating to 180°C for 3 h;

b Yields calculated by GC;

c Reaction conducted in a autoclave heating to 160°C for 3 h;

d Yield calculated by NMR;

e 10% in weight.

**Scheme 6:**
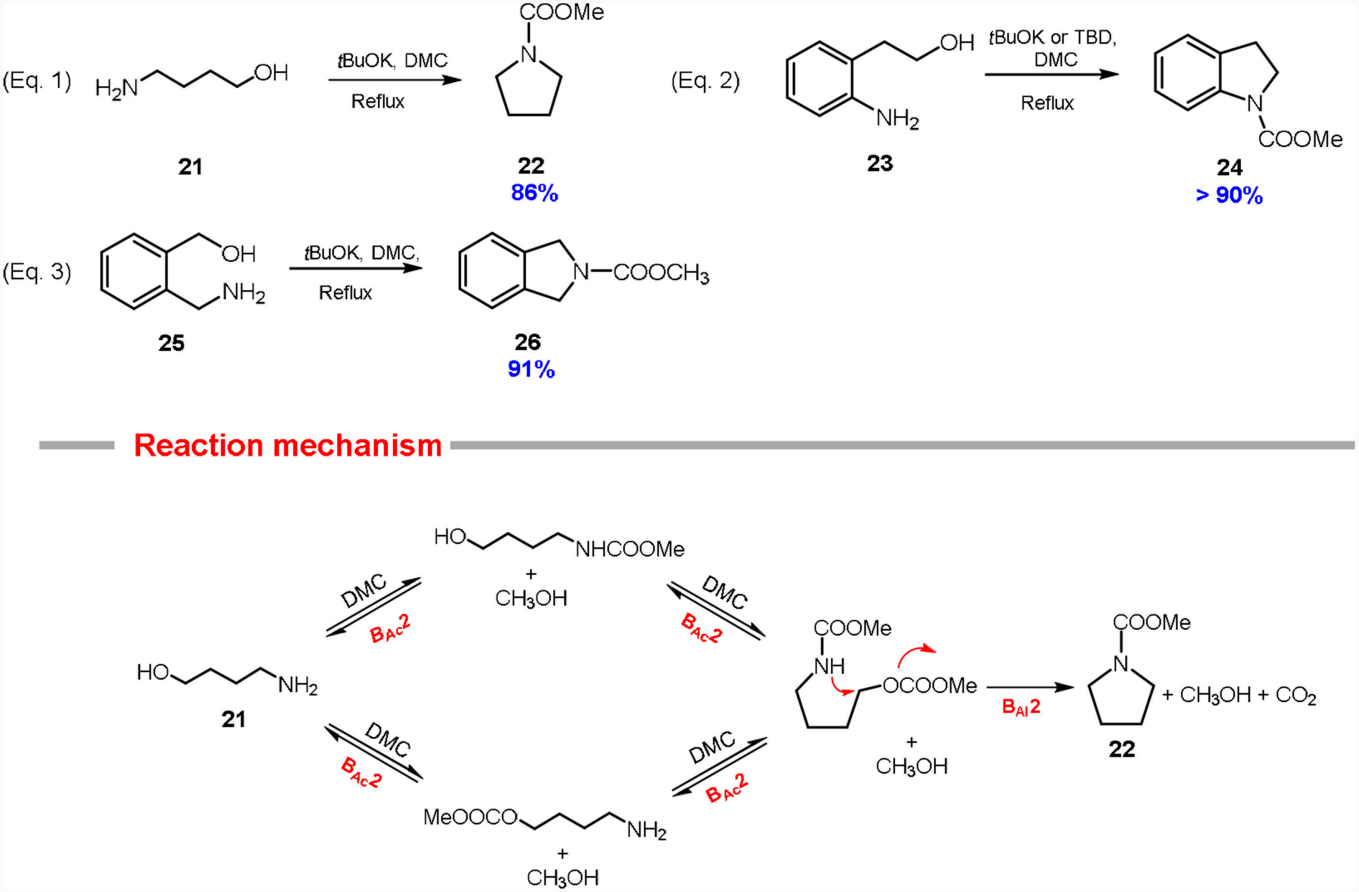
Preparation of pyrrolidine **22**, indoline **24** and isoindoline **26** via DMC chemistry. Reaction conditions: Equations 1, 3: substrates **21** and **25** (1.0 eq) were reacted with DMC (60.0 eq) and *t*BuOK (2.0–2.5 eq, at 90°C for 6 h. Equation 2: substrates **23** (1 eq) were reacted with DMC (60 eq) and *t*BuOK (2.5 eq) or TBD (0.5 eq), at 90°C for 6–21 h.

This cyclization reaction was also investigated at the reflux temperature of DMC (90°C), resulting in a high-yielding synthesis of the related pyrrolidine **22** (i.e., 86% isolated yield), although, in this case, an excess of potassium *tert*-butoxide (2.0 mol eq.) was required (Equation 1; Scheme 6).

The synthesis of pyrrolidine system via DMC chemistry is a remarkable example of Hard-Soft Acid-Base theory. In fact, the starting substrate includes two different nucleophiles, a primary amine and a primary alcohol capable to discriminate between the two electrophilic centers of the DMC to form the related pyrrolidine **22** in a one-pot cyclization. Most probably, the 4-amino-1-butanol **21** firstly undergoes carboxymethylation at the hydroxy (or the amine) group, then also the amine (or the hydroxy) group is carboxymethylated (B_Ac_2). As a consequence, the amino group of the so formed carbamate is softer in character and it undergoes fast alkylation to give selectively the carboxymethyl pyrrolidine. The formation of the cyclic compound is favored also because all the methoxycarbonylation reactions are equilibria, meanwhile the final cyclization reaction is kinetically driven (Reaction mechanism; Scheme 6).

2-(2-Aminophenyl)ethanol **23** and 2-(aminomethyl)benzyl alcohol **25** were also tested as substrates for the preparation of the related *N*-carboxymethyl indoline **24** and *N*-carboxymethyl isoindoline **26**, respectively (Equations 2, 3; Scheme 6). In particular, 2-(2-aminophenyl)ethanol **23** readily cyclized in high yield (> 90%) either at reflux temperature (90°C) in the presence of an excess of potassium *tert*-butoxide (2.5 mol eq.) or in autoclave (180°C) with a substoichiometric amount of the same base (0.1 mol eq.) (Equation 2; Scheme 6).

In addition, *N*-carboxymethyl indoline **24** can be obtained in quantitative yield (97%) at 90°C also employing a substoichiometric amount of TBD (0.5 mol eq.), although the reaction required 21 hours to reach the completion (Equation 2; Scheme 6) (Aricò et al., 2015a).

2-(Aminomethyl)benzyl alcohol **25** was efficiently converted into *N*-carboxymethyl isoindoline **26** (Equation 3; Scheme 6) in excellent yield (91%) when the reaction was conducted at reflux temperature in the presence of potassium *tert-*butoxide (2.5 mol eq.) and in a good yield (71%) when the cyclization was performed in autoclave using a substoichiometric amount of the same base (10% mol eq.).

Cyclisation of aliphatic and aromatic 1,4-amminoalcohols via DACs chemistry represents a good example of green chlorine-free approach to the related 5-membered-nitrogen heterocycles. These syntheses resulted overall high yielding, although the complexity of the substrates incorporating two different nucleophilic groups led to the use of an excess of base. Furthermore, the reaction mechanism has yet investigated in detail.

## 6-Membered Heterocycles

### 1,4-Dioxanes

The synthesis of six-membered heterocycle 2,3-dihydrobenzo[b]-[1,4]dioxine **28** was recently reported using DMC. Benzodioxanes are key structural units in numerous pharmaceuticals; examples include piperoxan, fluparoxan, and americanol A (Bao et al., 2008; Bagnoli et al., 2013). Isovanillyl sweetening agents also incorporate a 2,3-dihydro-1,4-benzodioxane unit. In addition, this ring is included in numerous natural products, such as silybin, isosilybin, haedoxan A, and eusiderin.

**Scheme 7:**
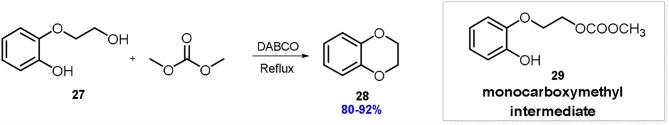
Preparation of 2,3-dihydrobenzo[b]-[1,4]dioxine **28** via DMC chemistry. Reaction conditions: **29**: DMC: DABCO in 1.00: 8.00: 0.05–1.00, at 90°C for 2–15 h.

In particular 2,3-dihydrobenzo[b]-[1,4]dioxine **28** can be easily prepared starting from commercially available 2-(2-hydroxyethoxy)phenol **27** by employing DMC both as reagent and reaction solvent in the presence of stoichiometric amounts of DBU, DABCO or TBD (Scheme 7) (Aricò et al., 2015a).

Among the organocatalysts investigated, DABCO (1.0 eq. mol) resulted very efficient (reaction time 2 h) in promoting the cyclization even when it was used in catalytic amounts (5% mol eq.). In fact, in the latter conditions 2,3-dihydrobenzo[b]-[1,4]dioxine **28** was isolated in 83% by simple removal of the exceeding solvent and without the use of any time-consuming column chromatography.

On the other hands, the reactions in the presence of DBU or TBD required longer reaction time and the experiment conducted in the presence of DBU showed lower selectivity toward the cyclic product, due to the presence of a reaction intermediate, the methoxycarbonyl derivative (Scheme 7).

The synthesis of 1,4-dioxan via DMC chemistry is an extension of the work reported on 5-membered cyclic compounds and it shows that the use of DMC as a sacrificial molecule in cyclization reaction can be employed also to more complex structures.

### Piperidines

The preparation of piperidine via DACs chemistry is mostly related to our recent studies on mustard carbonates (Aricò and Tundo, 2016c). These compounds are carbonate analogs of mustard gases, which are well-known vesicant and blistering agents used in several chemical warfares (Wang et al., 2012).

**Figure 3:**
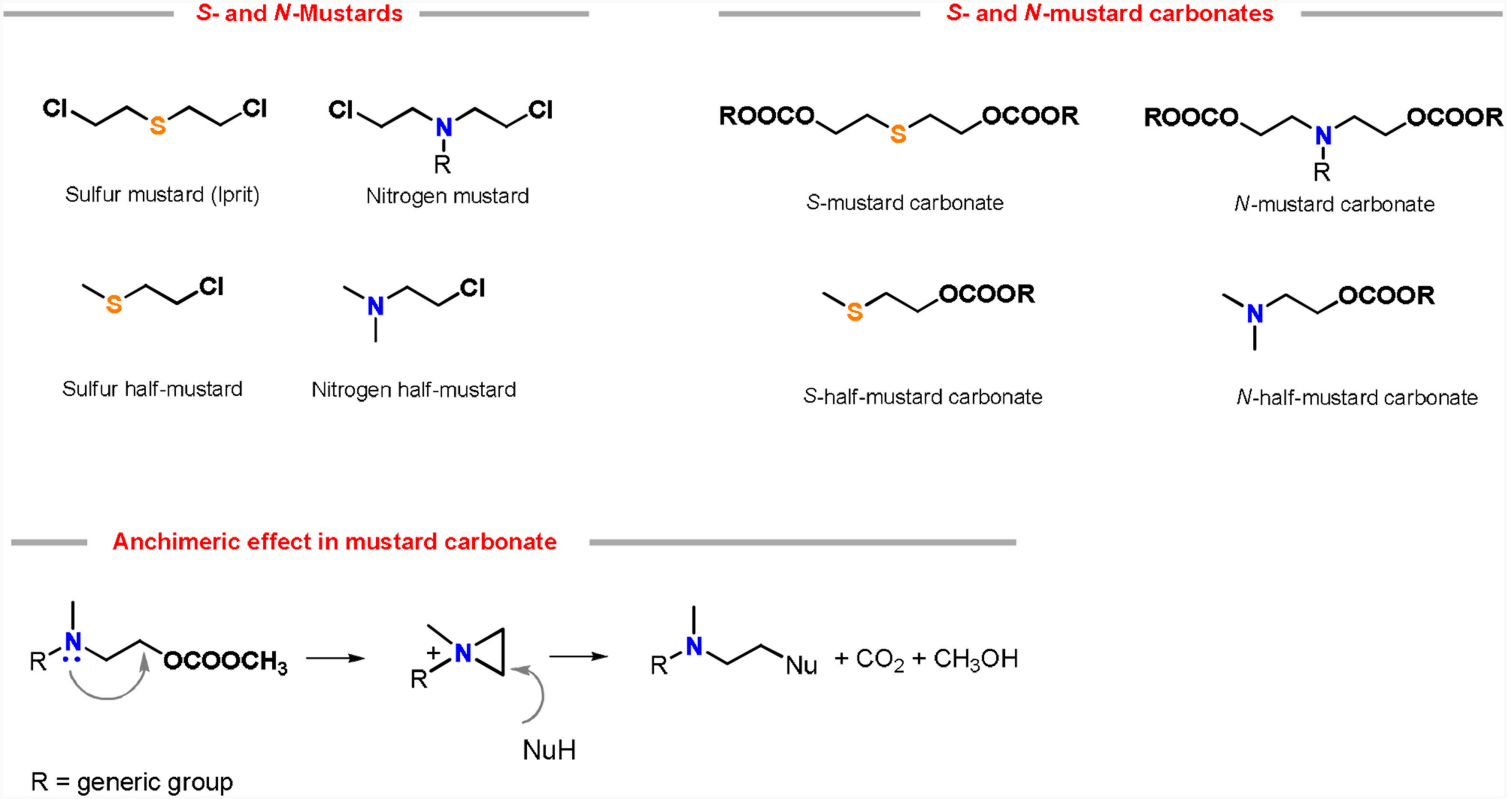
Chemical structures of mustard gases and their analog carbonates. Reactivity of nitrogen mustard carbonates in the presence of a nucleophile (NuH) via anchimeric effect.

It has been discovered that the substitution of the mustard gas chlorine atom with a carbonate moiety via DACs chemistry resulted in molecules showing a similar reactivity and kinetic behavior of their chlorine homologs, without sharing their toxicological profile (Aricò et al., 2012a) (Figure 3).

Sulfur and nitrogen mustard carbonates reactivity was investigated in the presence of numerous nucleophiles, both in autoclave at 180°C in absence of any base (Aricò et al., 2013) and in neat at 150°C using a substoichiometric amount of a K_2_CO_3_ as a base (Aricò et al., 2014). Reaction mechanism, effect of the leaving group and kinetics confirmed that these compounds retain the anchimeric effect of their mustard gas analogs (Aricò et al., 2016a). Besides, nitrogen mustard carbonates have also been used in the synthesis of azacrowns and polycarbonates (Aricò et al., 2015b).

**Scheme 8:**
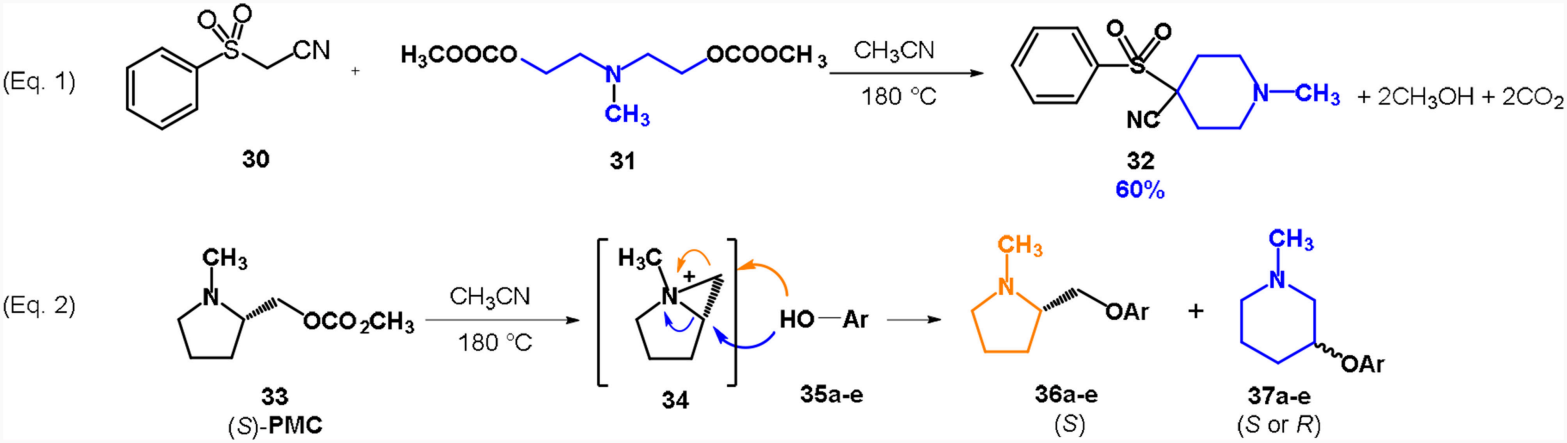
Preparation of piperidines via DACs chemistry by reaction of a symmetrical mustard carbonate with a CH_2_-acidic compound (Equation 1) or via ring expansion reaction of a pyrrolidine-based carbonate (Equation 2). Reaction conditions of Equation 1: **31**: **32** in 1: 1 molar ratio, in ACN, heated in a autoclave to 180°C for 7 h.

Two possible synthetic methodology for the synthesis of piperidines have been reported employing nitrogen-mustard carbonates: (i) by reaction of a symmetrical mustard carbonate with a CH_2_-acidic compound or (ii) via ring expansion reaction of a pyrrolidine-based carbonate (Scheme 8).

In the first case a methylene acidic molecule, i.e., phenylsulfonyl acetonitrile **30** was reacted with a symmetrical nitrogen mustard carbonate, namely bis-*N,N*-[(2-methylcarbonate)ethyl]-methylamine **31**, in an autoclave at 180°C in the absence of any base (Equation 1; Scheme 8). Surprisingly, the substrate underwent a double intermolecular cyclization (B_Al_2) leading to the formation of a 4-substituted piperidine **32** as the major product (Aricò et al., 2013). The pure piperidine can be isolated by a quick column chromatography in 60% yield. This reaction is a remarkable example of an intermolecular cyclization proceeding through a double alkylation reaction. Although several other substrates have been then exploited—i.e., phenylacetonitrile, bis(phenylsulfonyl)methane, 1,3-cyclohexanedione, ethyl acetoacetate, dimethyl malonate, etc.–, high selectivity toward the related piperidines was achieved only with phenylsulfonyl acetonitrile **30**.

The second approach to obtain a piperidine ring is related to the preparation of optically active 3-substituted piperdines, that are very common building blocks both in natural and bioactive compounds (Viegas et al., 2004; Castro et al., 2008).

A pyrrolidine-based mustard carbonate **33** (PMC) was prepared by reaction of (*S*)-1-methyl-2-pyrrolidinemethanol with DMC. The resulting compound was enantiomerically pure as confirmed by NMR investigation with a europium shift reagent.

The reaction of PMC **33** with a generic nucleophile, such as phenol **35a**, can lead to three products, i.e., a substituted (*S*)-pyrrolidine (**36a**) and the two enantiomers of a piperidine (**37a**) formed via ring expansion (Equation 2; Scheme 8 and Entry 1; Table 1). In fact, the stereogenic center of the pyrrolidine can be affected only by the nucleophilic attack on the more sterically hindered tertiary carbon of the bicyclic aziridinium intermediates (Aricò et al., 2018a).

Experiments were conducted with different substrates, such as phenol (**35a**), *p*-bromo (**35b**) and *p*-nitro (**35c**), *p*-cyanophenol (**35d**), 2-naphthol (**35e**) (Table 2). It was reported that less acidic nucleophiles, i.e. phenol **35a**, *p*-bromophenol **35b**, and 2-naphthol **35e** led mainly to substituted pyrrolidines **36a**, **36b**, and **36e**, respectively (Entry 1, 2, and 5; Table 2), whereas more acidic ones, *p*-nitro **35c** and *p*-cyanophenol **35d** formed preferably the related 3-substituted piperidines **37c** and **37d**. These results were ascribed to the fact that less acidic nucleophiles follow a kinetically controlled mechanism; on the other hand, more acidic substrates undergo a thermodynamically controlled mechanism, leading to the ring expansion reaction of the starting pyrrolidine.

**Table 2:** Pyrrolidine ring expansion by reaction with different nucleophiles^a^.

**Entry**	**Nucleophile**	**Yield (%)**	
		** 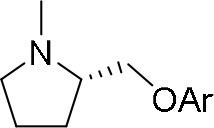 **	** 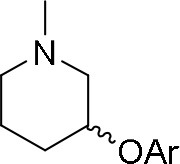 **
		**36a−e**	**36a−e**
1	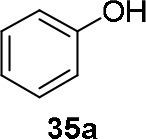	46	32
2	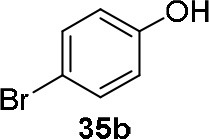	56	37
3	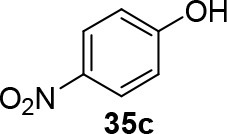	11	89
4	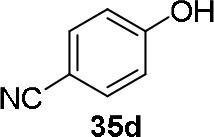	23	56
5	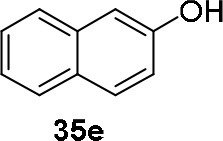	35	23

a Reaction conditions: ***33***: ***35a-e*** in 1: 1 molar ratio, in ACN heated in a autoclave to 180°C for 5–24 h.

The observed ring expansion of *N*-alkylated prolinols in the presence of acidic substrates is generally highly stereoselective: the resulting optically active 3-piperidines were achieved in high yields (up to 89% for *p*-nitrophenol **35c**). X-ray diffraction analyses demonstrated that this reaction is stereoselective with inversion of configuration. It is remarkable that this reaction is the first example of ring expansion of *N*-alkylated prolinols based on carbonate chemistry.

In both the reported examples for the preparation of piperidine via DACs chemistry, it is evident that the cyclization is driven by the presence of the anchimeric effect of the mustard carbonate and by the ability of the methoxycarbonate moiety to act as an efficient leaving group. The yield of the cyclization depends on the substrate employed. Further investigations are needed to increase the greenness of these reaction as in selected examples, a column chromatography was required to isolate the resulting piperdines.

### Cyclic Carbamates

Six-membered cyclic carbamates have numerous applications as pharmaceuticals, due to their biological activity mostly related to treatment of diseases connected to kinases activity (Hongqi and Gongchao, 2016). However, these cyclic carbamates also found application as herbicides with excellent crop-weed selectivity (Hino et al., 2009) and as monomers for the preparation of hyperbranched polyamines (Voit and Lederer, 2009) and polyurethane by cationic ring-opening polymerization (ROP) (Kreye et al., 2013).

Most of the synthetic routes to 1,3-oxazinan-2-ones involves phosgene or its derivatives (Murdock, 1968), alkyl halide chemistry (Trifunovic et al., 2010) and isocyanate compounds (Shibata et al., 1989). In the literature, there are also other procedures that generally require complex starting materials or multiple-step pathways, in order to achieve the final product (Mangelinckx et al., 2010).

**Scheme 9:**
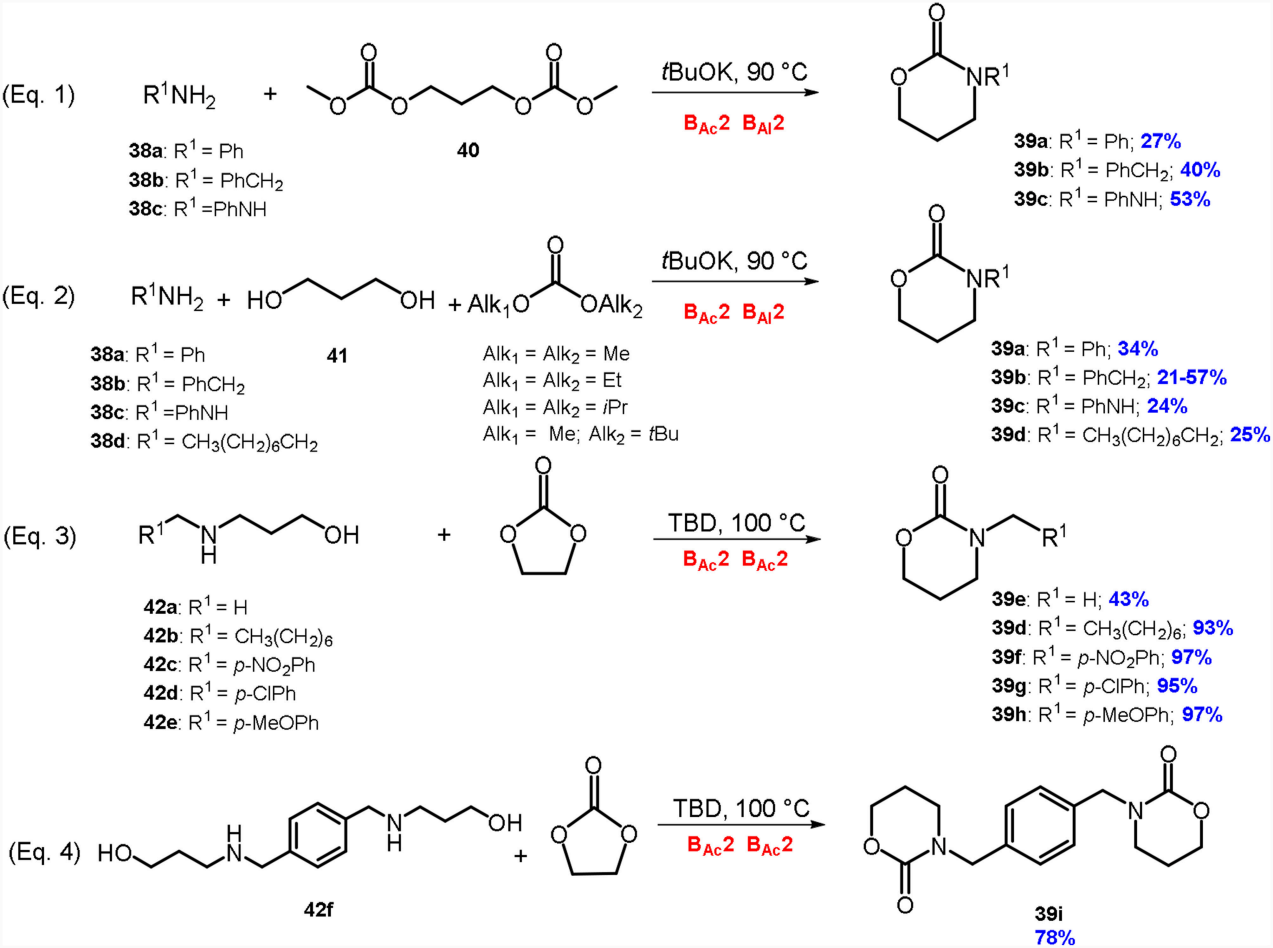
Synthesis of 1,3-oxazin-2-ones via DACs chemistry by reaction of: an amine with a dicarbonate derivative of 1,3-diols (Equation 1), an amine with a 1,3-diol and a dialkyl carbonate (Equation 2), 3-amino-1-propanols with ethylene carbonate (Equation 3); synthesis of an aryl bis(1,3-oxazinan-2-one) (Equation 4). Reaction conditions: Equation 1: **38a-c**: **40**: *t*BuOK in 1.0: 1.0: 1.0 molar ratio, at *T* = 90°C for 2 h; Equation 2: **38a-d**: **41**: DAC: *t*BuOK in 1.0: 1.0: 2.0: 3.0 molar ratio, at *T* = 90°C for 2 h; Equations 3, 4: **42a-f**: EC: TBD in 1.0: 1.0: 0.2 molar ratio, at *T* = 100°C, for 5–97 h.

Over the years, DACs chemistry has demonstrated to be very efficient for the preparation of differently substituted 1,3-oxazinan-2-ones. As an example, the reaction of primary amines **38a-c** with a dimethylcarbonate derivative of 1,3- propanediol **40** in the presence of a strong base led the formation of 6-memebered cyclic carbamates **39a-c** (Equation 1; Scheme 9).

In the best-found reaction conditions, a mixture of an amine (**38a-c**), dimethylcarbonate derivative of 1,3-propandiol **40** and potassium *tert-*butoxide (1:1:1 mol ratio) were reacted at 90°C to give the wanted 1,3-oxazinan-2-one (**39a-c**) (McElroy et al., 2012). Several amines were used as substrates including aniline **38a** benzylamine **38b** and phenylhydrazine **38c** leading to the formation of the related cyclic carbamate **39a-c** in modest yield (27–53%).

Attempts to achieve 1,3-oxazinan-2-ones **39a-d** through a one-pot synthesis were then conducted by reacting an amine **38a-d**, a 1,3-diol **41** and a dialkyl carbonate in the presence of a strong base (Equation 2; Scheme 9). In a typical procedure, benzylamine **38b** was reacted with 1,3-propandiol **41** in the presence of different DACs to give the related 1,3-oxazinan-2-one **39b** (McElroy et al., 2012). The plan was to perform the cyclization reaction in one-step by synthesizing the dialkylcarbonate derivative of 1,3-propandiol *in-situ*, through a cascade reaction. Following this procedure, six membered cyclic carbonates formed with modest yield (21-57%), depending on the substrate, the DAC used and the nature of the diol.

It was found that the more hindered the diol used, the lower the yield of 1,3-oxazinan-2-one. Conversely, a very high level of selectivity was achieved when a 1,3-diol with both primary functionalities were employed. It can be concluded that for cyclization to occur, a primary CH_2_ functionality (in α to the carbonate) must be present.

Regarding the effect of the DAC used, it was observed that the yield of cyclization increases by increasing the steric hindrance of the dialkyl carbonate used in the reaction. When DMC or diethylcarbonate (DEC) were employed, 3-benzyl-1,3-oxazinan-2-one **39b** was isolated in 21 and 36% yield respectively. Instead, when the reactions were carried out employing more sterically hindered diisoproprylcarbonate (DiPrC) or methyl *tert*-butyl methylcarbonate (M*t*BuC), the oxazinanone was isolated in higher yields (47 and 57% respectively).

These results are in good accordance with our previous investigations comparing the reactivity of amines and alcohols with DACs. The reaction of an amine with a sterically hindered DAC leads to the related carbamate in high yield as more hindered alkoxides resulted to be better leaving groups (Tundo et al., 2010b). Conversely, when an alcohol is reacted with a DAC in basic conditions the trend of the leaving group is almost the opposite of that observed with an amine (Tundo, McElroy and Aricò, 2012).

The general applicability of the one-pot synthesis to 1,3-oxazinan-2-ones, was studied employing several nucleophiles i.e., aniline **38a**, phenylhydrazine **38c**, and *n*-octylamine **38d**. Results showed a higher reactivity of the aromatic substrates.

The above discussed DACs-based synthetic approaches to cyclic carbamate (Equation 1-2; Scheme 9) were identified by EATOS software and Andraos spreadsheets analysis as the most promising on the basis of their low environmental impact (Toniolo et al., 2014). However, it should be mentioned that in both procedures, the formation of cyclic carbamates occurs together with several concurrent reactions leading to numerous by-products, such as aromatic carbamates, aromatic ureas and aliphatic and aromatic carbonates. Therefore, although cyclic carbamates are achieved as major products, a purification by column chromatography on silica gel is needed to isolate them.

Recently, our research group has reported a simpler and high yielding approach to cyclic carbamates **39d-i** by reaction of 3-amino-1-propanols **42a-f**, easily prepared by reductive amination, with ethylene carbonate, in the presence of catalytic amount (up to 5% mol eq.) of bicyclic nitrogen base TBD (Equation 3-4; Scheme 9). The procedure resulted in a general application for the synthesis of aliphatic and aromatic 1,3-oxazin-2-ones (Aricò et al., 2016b). In fact, several *N*-substituted 1,3-aminopropanols **42b-f** were synthesized and investigated as substrates; the related 1,3-oxazin-2-one derivatives **39d** and **39f-i** were achieved in good yield, without any further purification (78-98%). Noteworthy, this synthetic approach proved to be effective also in the synthesis of an aryl bis(1,3-oxazinan-2-one). This compound was achieved by a double intermolecular cyclization and isolated as a pure product in 78% yield (Equation 4; Scheme 9).

Compared to other previously reported DACs-based reactions, this intermolecular cyclization follows a double B_Ac_2 reaction mechanism, instead of a B_Ac_2 followed by a B_Al_2 mechanism. Furthermore, the ethylene carbonate has a double role being employed both as solvent and reagent in the synthesis of the related cyclic carbamate.

The main advantages of this synthetic approach are: simple work-up of the reactions, high yield, sustainable reaction conditions, general application and absence of by-products formation.

## Conclusions

The synthesis of heterocycles via DACs chemistry is a striking example of the versatility of this class of compounds. Following are reported the main results for each class of heterocycles investigated via DACs chemistry.

### 5- and 6-Membered *O*-Heterocycles

In the best-found reaction conditions, aliphatic and aromatic 1,4-diols gave the related heterocycles by reaction with DMC at reflux temperature (90°C) in the presence of catalytic amounts of superbases DBU or TBD. The cyclization reaction proceeds via B_Ac_2 mechanism followed by an intramolecular alkylation, i.e., B_Al_2 mechanism.

This green approach to synthesize tetrahydrofuran systems does not use any additional solvent other than the selected DAC and the wanted cyclic products can be isolated as pure by a simple filtration on a silica pad.

6-member *O*-heterocycle 1,4-benzodioxane was also prepared in quantitative yield by a similar approach demonstrating the possibility to use this methodology with more complicated substrates.

In this view, industrially relevant compounds, isomannide, isosorbide, and ambroxan were achieved in quantitative yield starting from D-mannitol, D-sorbitol and amberlyn diol respectively via DMC-promoted cyclization. Besides, in all cases, the chiral integrity of the substrate was preserved.

In a specific case, the synthesis of 2,3-benzofuran, the cyclization reaction was conducted in acidic conditions. In this case DMC acts as methoxycarbonylation agent and leaving group in the intramolecular cyclization, which is promoted by the formation of a phenonium ion.

### 5-(Hydroxymethyl)Furfural

In the case of bio-based platform chemical HMF, DMC was used as a very efficient extracting solvent, leading to one of the very few procedure in which HMF can be easily separated from the reaction mixture and isolated as a pure product (92% isolated yield). This synthetic approach was evaluated according to several green metrics and resulted the most promising in terms of mass consumption.

### 5-Membered *N*-Heterocycles

Pyrrolidines, indolines, and isoindolines were also achieved using DMCs as sacrificial molecules. In this case, the starting substrates−1,4-aminoalcohols–incorporates two different nucleophilic groups, thus the cyclization requires an excess of a base and, for specific products, the products purification is also necessary.

### Piperidines

For this class of heterocycles the DACs-based synthetic approach took advantage of the anchimeric effect of a new family of compounds, named mustard carbonates. In particular, 4-substituted piperidines were prepared by reaction of compounds including acidic CH_2_ groups, in the presence of a symmetrical nitrogen mustard carbonate. However, it should be mentioned that this synthetic approach resulted high yielding only in the case of phenylsulfonyl acetonitrile.

Optically active 3-piperidines can be efficiently synthesized via ring expansion of methylcarbonate derivatives of *N*-alkylated prolinols in the presence of acidic phenols. In this synthetic approach, the anchimeric effect of the prolinol derivative is pivotal for the high yielding ring expansion reaction (up to 89 % for *p*-nitrophenol). It should be mention that in some cases the heterocycle purification was necessary.

### 6-Membered Cyclic Carbamates

Numerous examples of 1,3-oxazinan-2-ones were synthesized via DACs employing different synthetic approaches, such as by reaction of an amine with a dicarbonate derivative of 1,3-diols or by reaction of an amine with a 1,3-diol and a dialkyl carbonate. These two methodologies are based upon a B_Ac_2 mechanism followed by an intramolecular alkylation reaction (B_Al_2 mechanism). Despite being identified by EATOS software and Andraos spreadsheets analysis as the most promising cyclic carbamate preparation, both procedures lead to the formation of numerous by-products. Recently a more efficient and greener procedure was reported and it was based upon the reaction of easily prepared *N*-substituted 3-amino-1-propanol derivatives with ethylene carbonate. In this preparation EC is used as a carbonylating agent and the 6-membered cyclic carbamates are formed via a double B_Ac_2 mechanism.

In conclusion, over the last 10 years several examples of green synthesis of heterocycles have been reported via DACs chemistry. It is noteworthy that, in these cyclizations, DMC or other DACs act as reaction media and as a sacrificial molecule, similarly to chlorine compounds. Although chlorine compounds employed in cyclization reactions generally are effective at lower temperature, in the case of DMC (as an example), the intrinsic toxicity of chlorine-based molecules is avoided since at the end of the reaction DMC is fully converted into methanol and CO_2_.

## Future Perspectives

The green synthesis of heterocycles via DACs chemistry is still relatively new and unexplored, thus in the near future, it is expected that DACs might be employed for the preparation of more complex cyclic compounds. Future exploitations might include heterocycles incorporating more heteroatoms, such as benzoxazine and benzothiazine. It would be also interesting to extend DACs-based heterocyclic to the synthesis to seven- and eight-membered heterocycles to prove the robustness of this approach.

Finally, an example of macrocycle preparation via DACs chemistry has already been explored (Aricò et al., 2015b) and new investigations in the preparation of macromolecules could also take in consideration.

## Author Contributions

All authors listed have made a substantial, direct and intellectual contribution to the work, and approved it for publication.

### Conflict of Interest Statement

The authors declare that the research was conducted in the absence of any commercial or financial relationships that could be construed as a potential conflict of interest.
